# Development and validation of an eDNA protocol for monitoring endemic Asian spiny frogs in the Himalayan region of Pakistan

**DOI:** 10.1038/s41598-022-09084-1

**Published:** 2022-04-04

**Authors:** Muhammad Saeed, Muhammad Rais, Ayesha Akram, Maggie R. Williams, Kenneth F. Kellner, Syed A. Hashsham, Drew R. Davis

**Affiliations:** 1 Herpetology Lab, Department of Wildlife Management, Faculty of Forestry, Range Management and Wildlife, Pir Mehr Ali Shah, Arid Agriculture University Rawalpindi, Rawalpindi, 46000 Pakistan; 2 Department of Civil and Environmental Engineering, Michigan State University, East Lansing, MI USA; 3 School of Engineering and Technology, Institute for Great Lakes Research, Central Michigan University, Mount Pleasant, MI USA; 4 Global Wildlife Conservation Center, State University of New York College of Environmental Science and Forestry, Syracuse, NY USA; 5 Center for Microbial Ecology, Michigan State University, East Lansing, MI USA; 6 Department of Plant, Soil, and Microbial Sciences, Michigan State University, East Lansing, MI USA; 7 School of Earth, Environmental, and Marine Science, The University of Texas Rio Grande Valley, Brownsville, TX USA; 8 Biodiversity Collections, Department of Integrative Biology, The University of Texas at Austin, Austin, TX USA

**Keywords:** Ecology, Genetics

## Abstract

Wildlife monitoring programs are instrumental for the assessment of species, habitat status, and for the management of factors affecting them. This is particularly important for species found in freshwater ecosystems, such as amphibians, as they have higher estimated extinction rates than terrestrial species. We developed and validated two species-specific environmental DNA (eDNA) protocols and applied them in the field to detect the Hazara Torrent Frog (*Allopaa hazarensis*) and Murree Hills Frog (*Nanorana vicina*). Additionally, we compared eDNA surveys with visual encounter surveys and estimated site occupancy. eDNA surveys resulted in higher occurrence probabilities for both *A. hazarensis* and *N. vicina* than for visual encounter surveys. Detection probability using eDNA was greater for both species, particularly for *A. hazarensis*. The top-ranked detection model for visual encounter surveys included effects of both year and temperature on both species, and the top-ranked occupancy model included effects of elevation and year. The top-ranked detection model for eDNA data was the null model, and the top-ranked occupancy model included effects of elevation, year, and wetland type. To our knowledge, this is the first time an eDNA survey has been used to monitor amphibian species in the Himalayan region.

## Introduction

Wildlife monitoring programs are crucial for the assessment of species and habitats and for the management of factors affecting them^1^. This is particularly important for species inhabiting freshwater ecosystems, such as amphibians, as they have higher estimated extinction rates than terrestrial species^2^. Amphibians are among the most threatened group of vertebrates with 41% of assessed species being threatened with extinction^3^. Populations of amphibians are declining globally; however, amphibians inhabiting mountain streams at high elevations are facing particularly large declines in population size^4^. Amphibian decline is a multifaceted global problem with subtle underlying mechanisms in most instances^5,6^. Factors such as habitat degradation and loss^7–10^, disease^11,12^, ultraviolet radiation^13,14^, climate change^15–17^, competition with invasive species^18^, chemicals^19^, and synergistic effects of multiple stressors^20,21^ are known to impact various amphibian species around the world.

Endemism is considered a key criteria for monitoring and conservation strategies of species at national or regional levels^22^. In freshwater ecosystems, many endemic species evolved in limited geographic ranges^23,24^, often developing unique characteristics that help shape their ecosystems^25^. Given their unique traits and restricted distribution, the probability of extinction is high for endemic species^26^. The likelihood of extinction further increases when endemic species have poor dispersal ability. Since 1500, 51 of 62 (82%) extinct species of European animals were confined to one country or had a limited geographical distribution^27^. Likewise, endemic anurans with limited dispersal ability are feared to go extinct due to climate change^28^.

The success of any management program relies heavily on the selection and standardization of monitoring methods^29^, and selecting the optimal method for monitoring a given amphibian population can be challenging. A number of survey methods for amphibians are available, such as visual encounter surveys, area constrained searches, egg mass counts, tadpoles counts, pitfall traps, kick sampling, stovepipe sampling, and auditory sampling^30^. Traditional monitoring programs require financial resources, labor, expertise in species identification, and survey skills^31^. Alternatively, novel, non-invasive survey methods may require less labor, time, and cost, which can increase the efficiency of monitoring programs^32^. Adoption of a suitable statistical analysis approach for eDNA-based surveys is also crucial to make reliable inferences. Occupancy modeling is a powerful tool to estimate detection and site occupancy probabilities despite imperfect detection^33^. It is challenging to detect all species, populations, or individuals present within an area during a given survey^34,35^, which is often referred to as imperfect detection. Occupancy modeling requires multiple visits to a site to estimate the probabilities of occurrence when detection is imperfect. The pattern of detection/non-detection contains the information on detection probability, which allows one to calculate the true proportion of occupied sites^36,37^.

Environmental DNA (eDNA) has emerged as a complementary tool for the monitoring of invasive, threatened, rare, or endemic species of fish, amphibians, and other aquatic species. A major advantage of eDNA for monitoring is that it can be used with minimal damage to the monitored species and ecosystem^38–46^. eDNA methods have been successfully used to monitor many rare and threatened anurans, such as the Yellow-Legged Frog (*Rana sierrae*)^46^, Pool Frog (*Pelophylax lessonae*)^47^, Trinidad Golden Tree Frog (*Phytotriades auratus*)^48^, and Rocky Mountain Tailed Frog (*Ascaphus montanus*)^40^, when traditional methods have failed. Calling surveys are one of the most popular sampling methods for anurans because male vocalization to attract females is often easily identifiable and species-specific^49,50^, however, not all anurans produce noticeable vocalizations, and the use of eDNA-based survey methods may provide better detections of these species.

Here, we developed an eDNA-based approach to survey for the Hazara Torrent Frog (*Allopaa hazarensis*) and Murree Hills Frog (*Nanorana vicina*), two species endemic to montane regions of Pakistan. Males of both species do not produce noticeable breeding calls; therefore, visual encounter surveys have previously been used to monitor these species. Visual encounter surveys of these two species require multiple visits at night to result in successful detection, increasing cost, time, and effort in the field for large scale monitoring. We conducted this study: (1) to develop species-specific eDNA protocols and validate them in a laboratory setting; (2) to apply the protocol in the field to detect *A. hazarensis* and *N. vicina* and estimate site occupancy; and (3) to compare eDNA monitoring results with traditional visual encounter surveys. To our knowledge, this is the first time an eDNA assay has been used to monitor amphibian species in the Himalayan region. Although the methods we developed are specific to *A. hazarensis* and *N. vicina*, our study provides a useful framework for the development of eDNA survey methods and their application for species and population monitoring and conservation across a wide variety of taxa, particularly for montane freshwater streams.

## Results

### Development and validation of eDNA protocols

A ~ 550 bp region of the mitochondrial 16S rRNA gene sequence of *A. hazarensis* and *N. vicina* was obtained using Sanger sequencing. Based on the resulting alignment of sequences, five primer sets were designed targeting *A. hazarensis* (Allopaa1–5) and fourteen primers were designed targeting *N. vicina* DNA (Nanorana1–14; Table 1)*.* Three primer sets (Alloppa1–3) did not amplify *A. hazarensis* tissue DNA and were excluded from further assessment. Two primer sets (Allopaa4, Allopaa5) successfully amplified DNA and were selected for further evaluation. In the first approach (in silico testing), Allopaa4 and Allopaa5 did not match any non-target DNA. In the second approach (in vitro testing), primer sets Allopaa4 and Allopaa5 were tested against DNA from four sympatric anurans: *A. hazarensis*, *N. vicina*, Common Skittering Frog (*Euphylctis kalasgramensis*), and Indus Valley Bull Frog (*Hoplobatrachus tigerinus*)*.* The Allopaa5 primer set amplified DNA from *A. hazarensis* and *N. vicina*, but not *E. kalasgramensis* or *H. tigerinus* and was excluded from further evaluation. The Allopaa4 primer set was found to amplify the corresponding gene amplicon from *A. hazarensis* DNA, but not from the other three sympatric species tested*.* A third approach to evaluate the specificity involved comparative analysis with field data. The developed eDNA assay for *A. hazarensis* gave positive results from sites where the species was recorded using traditional methods and negative for samples collected from the Korang River (Islamabad Capital Territory), where the species is historically absent. A best-fit linear regression is represented by y = −3.38x + 21.79 (R^2^ = 0.99) was obtained for the data. The Allopaa4 primer set targeted small portion of the mitochondrial 16S rRNA gene (~ 50 bp) of *A. hazarensis* DNA, which was confirmed by gel electrophoresis and sequencing. Based on the sensitivity and specificity, the Allopaa4 primer set was considered the most appropriate among the five designed primer sets and used for subsequent eDNA monitoring for *A. hazarensis*.

**Table 1:** Details of primers used and designed in the current study.

Primer name	Sequence	Annealing temp. (°C)	Product length	Reference or source
16Sar-F	CGC CTG TTT AAC AAA AAC AT	60	550 +	Palumbi^87^
16Sar-R	CCG GTC TGA ACT CAG ATC ACG T			
Allopaa1-F	TCT GCC TGT TGG TTT TGG GT	60	839	This work
Allopaa1-R	CAC GTC CTC AGG AAC CAG TC			
Allopaa2-F	TAT CAA CGG CAT CAC GAG GG	60	929	This work
Allopaa2-R	CTG TTC ATC CCT GCG TTC CA			
Allopaa3-F	CCG TGA AGA AGC GGG GAT AA	60	880	This work
Allopaa3-R	TGC ATC GCT CCC TGT TCA TC			
Allopaa4-F	TAG CAC GAA AAT TCT GCC TGT	60	~ 50	This work
Allopaa4-R	TCC GTT CAT CGT GGA GGT TTA			
Allopaa5-F	CCA CAA AAT TCT GCA TAG CAC G	60	96	This work
Allopaa5-R	GTT AAT TCC GTT CAT CGT GGA G			
Nanorana 1-F	CGT GAA GAA GCG GGG ATG AA	60	437	This work
Nanorana 1-R	CGA CTC GTC AGC TGA GAA CA			
Nanorana 2-F	ACA GTG AGA CAA GCT ACG CA	60	103	This work
Nanorana 2-R	TGC AGC ATC TAC GTC AGC AC			
Nanorana 3-F	AGC TGA CGA GTC GAG TCA AA	60	211	This work
Nanorana 3-R	GCG ATC TGC AGA CAA CTA CG			
Nanorana -4 F	CAG CTG ACG AGT CGA GTC AA	60	149	This work
Nanorana -4 R	ATG CAG CAT CTA CGT CAG CA			
Nanorana -5 F	AGA CCC CAT GGA GCT TCA AA	60	454	This work
Nanorana -5 R	TGC GTA GCT TGT CTC ACT GT			
Nanorana 6-F	GAC CCC AGG CTA TGC AGA AT	60	114	This work
Nanorana 6-R	GGA AAA CTA TTT CCC CCG GA			
Nanorana 7-F	AAA CAG GAC CCC AGG CTA TG	60	123	This work
Nanorana 7-R	AAA GGA AAA CTA TTT CCC CCG GA			
Nanorana 8-F	ATT CTT AGG GTG GTG GCC CT	60	118	This work
Nanorana 8-R	ATT CTG CAT AGC CTG GGG TC			
Nanorana 9-F	GGG TGG TGG CCC TCA GAT TA	60	103	This work
Nanorana 9-R	TAG CCT GGG GTC CTG TTT GT			
Nanorana 10-F	GAC CCC ATG GAG CTT CAA AC	60	65	This work
Nanorana 10-R	GGG CCC CCG GTT TTT CAA			
Nanorana 11-F	CGT GAA GAA GCG GGG ATG AA	60	98	This work
Nanorana 11-R	GCC CCC GGG TAA GGA AAA T			
Nanorana 12-F	TGA AGA AGC GGG GAT GAA TCT	60	95	This work
Nanorana 12-R	CCC CCG GGT TAG CCA AA			
Nanorana 13-F	TGA AGA AGC GGG GAT GAA TCT A	60	96	This work
Nanorana 13-R	GCC CCC GGG TTA GCC A			
Nanorana 14-F	GTG AAG AAG CGG GGA TGA AT	60	99	This work
Nanorana 14-R	GGG CCC CCG GGT TAG			

Out of the 14 designed primer sets for *N*. *vicina*, two primer sets were selected (Nanorana8 and Nanorana9) by following the same validation steps used for *A. hazarensis*. After evaluation, Nanorana8, which targeted a ~ 90 bp portion of the 16S rRNA gene was found as most appropriate based on sensitivity and specificity. The selected primer set targeted only *N. vicina* DNA and did not amplify DNA of the other three non-target species (*A*. *hazarensis*, *E*. *kalasgramensis*, and *H*. *tigerinus*). A best-fit linear regression was represented by y = −3.50x + 25.27 (R^2^ = 0.99).

### Occupancy modeling: visual observation data

**Figure 1:**
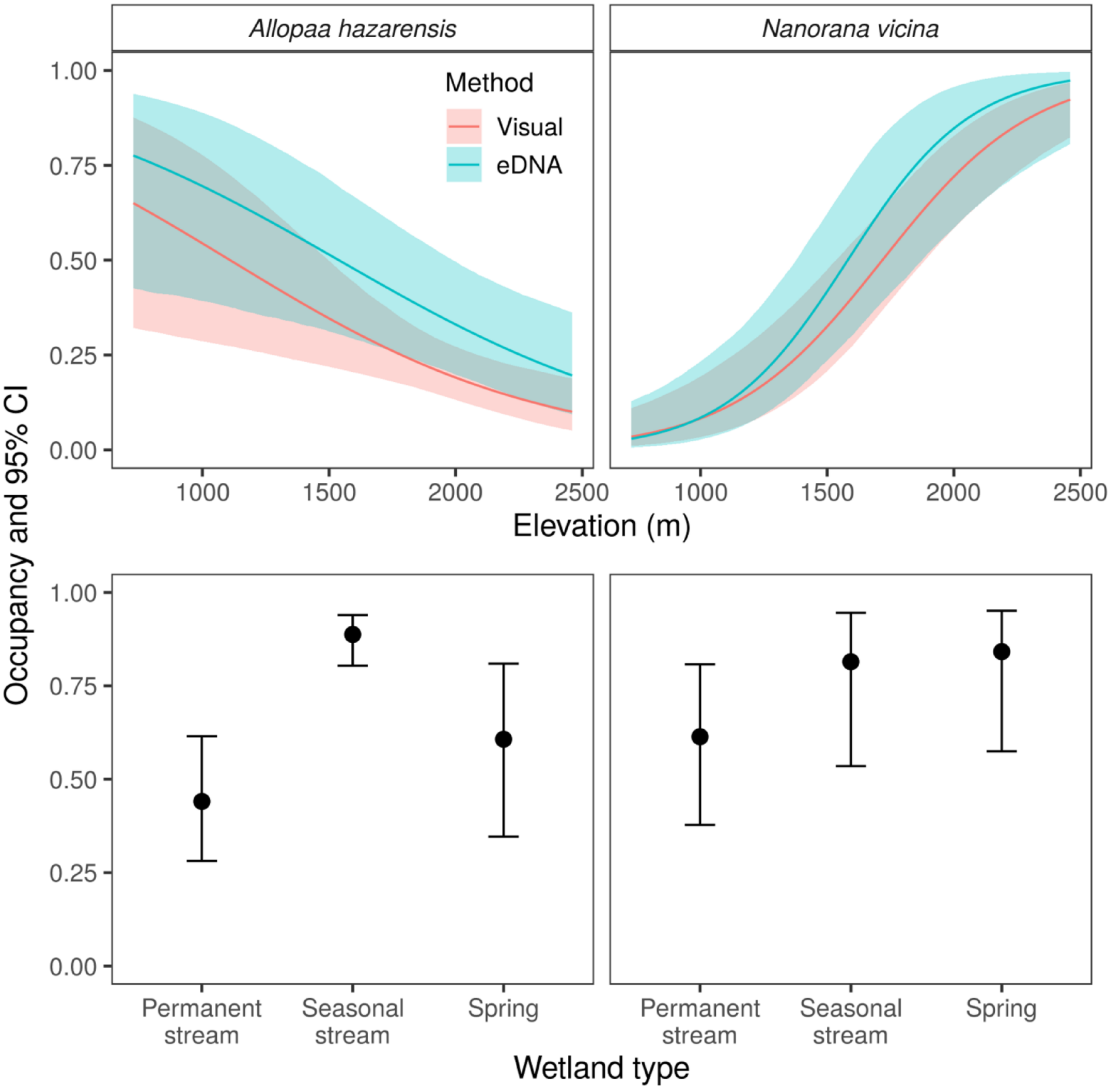
Top row: marginal effects of elevation on occupancy of the Hazara Torrent Frog (*Allopaa hazarensis*) and Murree Hills Frog (*Nanorana vicina*) based on multispecies occupancy models fit using visual observation and eDNA data. Bottom row: effect of wetland type on occupancy for the two frog species based on a multispecies model fit with eDNA data. Error bars and shaded areas represent 95% confidence intervals.

In total, we visually observed *A. hazarensis* 44 times out of 442 sampling occasions across 34 sites and two years. Naïve occurrence probability was 0.26 and 0.18 in years 1 and 2, respectively. We applied empirical Bayes methods using the top-ranked model to obtain estimates of the posterior distribution of latent (i.e., unobserved) occupancy states at each site and in each year^51^. Estimated proportion of sites occupied using this method was 0.28 (standard deviation [SD] = 0.019; 95% confidence interval [CI]: 0.26–0.32) in year 1 and 0.23 (SD = 0.052, 95% CI: 0.18–0.37) in year 2. We observed *N. vicina* 152 times out of 442 sampling occasions. Naïve occurrence was 0.50 and 0.44 in years 1 and 2, respectively. Model-based estimates of occupancy obtained using empirical Bayes methods were identical to the naïve estimates in both years. Thus, based on the model, *N. vicinia* was detected at least once at all sites where it was present. This result corresponds with the relatively high detection probability estimates for the species in each sampling occasion (> 0.6; see below), and the large number of sampling occasions (6–7). The top-ranked detection model for the visual observation data included effects of both year and temperature (Table 2). The estimated mean detection probability for *A. hazarensis* was 0.54 (95% CI: 0.40–0.67) and 0.31 (95% CI: 0.14–0.55) in years 1 and 2, respectively. For *N. vicina* detection probability was 0.67 (95% CI: 0.55–0.77) and 0.90 (95% CI: 0.81–0.94) in years 1 and 2, respectively. The top-ranked model for occupancy included effects of elevation and year for both *A. hazarensis* and *N. vicina* and did not include an interaction term (Table 3). Elevation had a significant negative effect on the occupancy of *A. hazarensis*, and a significant positive effect on occupancy of *N. vicina* (Table 4, Fig. 1). Though year was included in the top-ranked occupancy model, it was not significant (Table 4).

**Table 2:** Comparison of candidate models for the detection component of a multispecies occupancy model for the Hazara Torrent Frog (*Allopaa hazarensis*) and Murree Hills Frog (*Nanorana vicina*).

Model	Parameters	AIC		ΔAIC	Weight	
**Visual observation data**						
Year + Temperature	8	519.94		0.00	0.82	
Year	6	523.03		3.09	0.18	
Temperature	6	549.49		29.55	0.00	
Null	4	553.97		34.03	0.00	
**eDNA data**						
Null	4	679.85		0	0.78	
Year	6	682.36		2.51	0.22	

AIC = Akaike Information Criterion, ΔAIC = the difference in AICc values between the given model and the model that is most likely to have generated the data (i.e., the one with the lowest AICc), and is a relative measure conditional on the candidate set of models.

**Table 3:** Comparison of candidate models for the occupancy component of a multispecies occupancy model for the Hazara Torrent Frog (*Allopaa hazarensis*) and Murree Hills Frog (*Nanorana vicina*).

Model	Parameters	AIC	ΔAIC	Weight
**Visual observation data**				
Elevation + Year, No Species Interaction	12	492.19	0	0.80
Elevation + Year, Species Interaction	15	496.18	3.98	0.11
Elevation + Wetland + Year, No Species Interaction	16	496.54	4.35	0.09
Elevation + Wetland + Year, Species Interaction	21	504.37	12.18	0.00
Null, Species Interaction	9	528.82	36.62	0.00
**eDNA data**				
Elevation + Wetland + Year, No Species Interaction	12	636.30	0	0.90
Elevation + Year, No Species Interaction	8	642.22	5.92	0.05
Elevation + Wetland + Year, Species Interaction	17	642.59	6.3	0.03
Elevation + Year, Species Interaction	11	644.03	7.73	0.02
Wetland + Year, No Species Interaction	10	680.89	44.59	0.00

Only the top five models for each data type are presented. AIC = Akaike Information Criterion, ΔAIC = the difference in AICc values between the given model and the model that is most likely to have generated the data (i.e., the one with the lowest AICc), and is a relative measure conditional on the candidate set of models.

**Table 4:** Parameter estimates from the top-ranked (based on AIC) multispecies occupancy model for the Hazara Torrent Frog (*Allopaa hazarensis*) and Murree Hills Frog (*Nanorana vicina*) using visual observation data.

Species	Parameter	Estimate	SE	*Z*	*P*
*A. hazarensis*	Intercept	−0.95	0.26	−3.70	< 0.01
	Elevation	−0.87	0.29	−2.97	< 0.01
	Year 2	−0.37	0.31	−1.18	0.24
*N. vicina*	Intercept	−0.08	0.29	−0.27	0.78
	Elevation	1.78	0.29	6.20	< 0.01
	Year 2	−0.24	0.27	−0.89	0.37

Continuous covariates were standardized prior to analysis. The effect of year 2 is relative to a baseline of year 1. SE = Standard error, *P* = level of significance.

### Occupancy modeling: eDNA data

Using eDNA, we detected *A. hazarensis* 196 times out of 442 sampling occasions. Naïve occurrence probability was 0.53 in both years 1 and 2. Using the top-ranked eDNA model and empirical Bayes methods, model-based occupancy was identical to naïve occupancy in both years. This corresponded with very high detection probabilities in each sampling occasion (> 0.8, see below) and a large number of sampling occasions (6–7 visits). Thus, we likely detected *A. hazarensis* at all sites where it was present. We detected *N. vicina* 216 times out of 442 occasions. Naïve occurrence was 0.62 in both years 1 and 2. As with *A. hazarensis*, model-based occupancy in each year obtained using empirical Bayes methods was identical to the naïve estimate due to high detection probabilities and a large number of sampling occasions. The top-ranked detection model for eDNA data was the null model (Table 2). Estimated mean detection probability was 0.83 (95% CI: 0.77–0.89) for *A. hazarensis* and 0.79 (95% CI: 0.72–0.84) for *N. vicina*. The top-ranked occupancy model for the eDNA data included an effect of elevation, year, and wetland type; as with visual observation data, the top model did not include a two-species interaction term (Table 2). Elevation had a significant negative effect on the occupancy of *A. hazarensis*, and a significant positive effect on the occupancy of *N. vicina*, matching the results from the visual observation data (Table 5, Fig. 1). Seasonal streams had significantly higher occupancy than permanent streams for *A. hazarensis* (Table 5, Fig. 1), and both seasonal streams and springs had significantly higher occupancy than permanent streams for *N. vicina* (Table 5, Fig. 1). As with the visual observation model, year was in the top-ranked model but its effects were not significant (Table 5).

**Table 5:** Parameter estimates from the top-ranked (based on AIC) multispecies occupancy model for the Hazara Torrent Frog (*Allopaa hazarensis*) and Murree Hills Frog (*Nanorana vicina*) using eDNA data.

Species	Parameter	Estimate	SE	*Z*	*P*
*A. hazarensis*	Intercept	−0.24	0.38	−0.64	0.53
	Elevation	−0.82	0.29	−2.86	< 0.01
	Seasonal stream	2.30	0.33	6.95	< 0.01
	Spring	0.67	0.45	1.51	0.13
	Year 2	−0.02	0.36	−0.04	0.97
*N. vicina*	Intercept	0.46	0.50	0.94	0.35
	Elevation	2.18	0.52	4.16	< 0.01
	Seasonal stream	1.02	0.49	2.07	0.04
	Spring	1.20	0.46	2.62	< 0.01
	Year 2	0.05	0.41	0.11	0.91

Continuous covariates were standardized prior to analysis. The effects of “Seasonal stream” and “Spring” are relative to a baseline of permanent stream. The effect of year 2 is relative to a baseline of year 1. SE = Standard error, *P* = level of significance.

### Comparison between eDNA and visual encounter surveys methods

**Figure 2:**
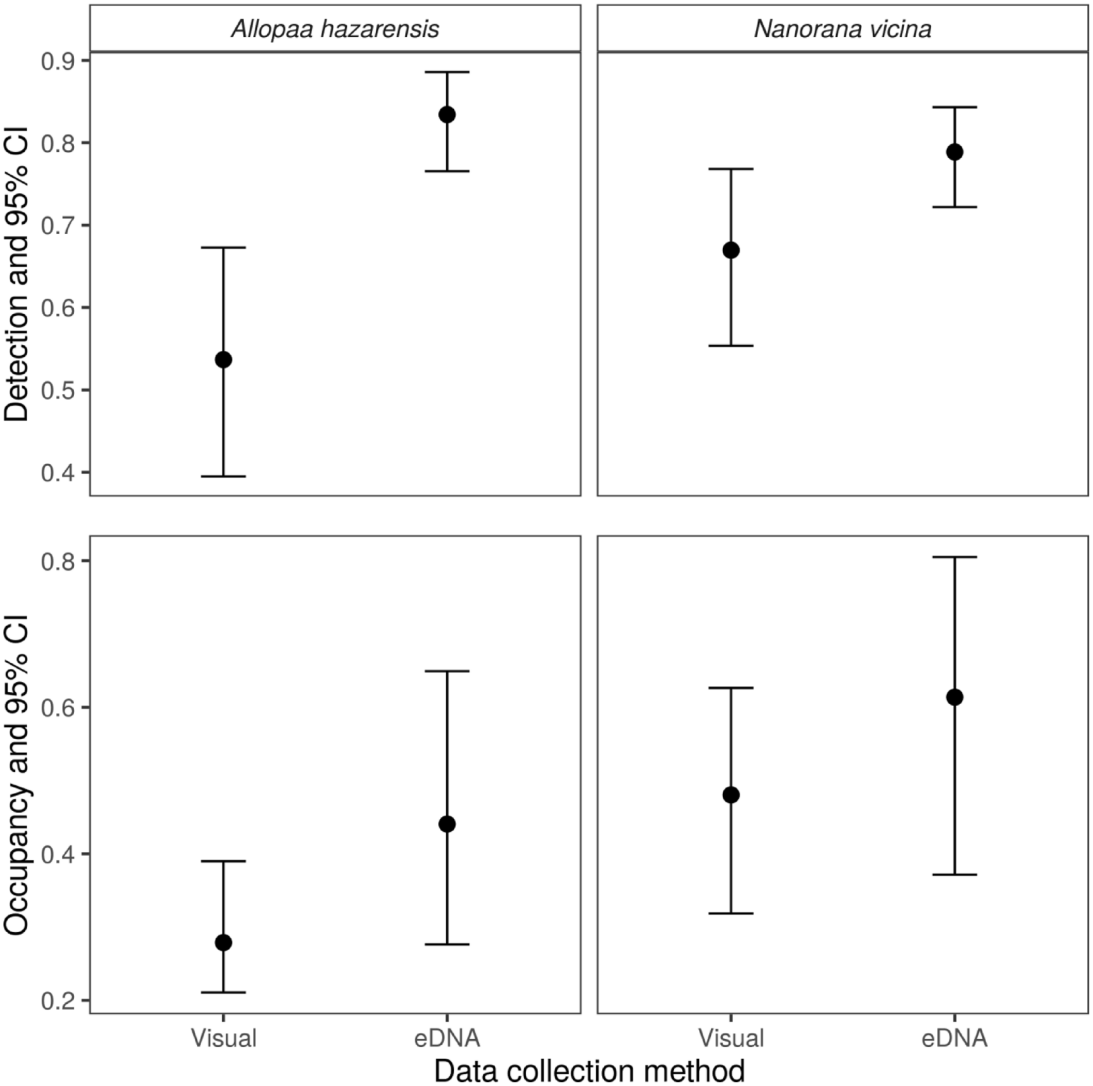
Comparison of occupancy probability and detection probability estimates (and 95% confidence intervals) from multispecies occupancy models of the Hazara Torrent Frog (*Allopaa hazarensis*) and Murree Hills Frog (*Nanorana vicina*) fit using visual observation and eDNA data. Detection probabilities from the visual observation model were calculated for year 1 and the mean temperature. Occupancy probabilities were calculated for year 1 and the mean elevation (both models) and at the permanent stream wetland type (eDNA model).

Detection probability using the eDNA assay was greater for both species, particularly for *A. hazarensis* (Fig. 2). Estimated occupancy probability was also higher using eDNA data relative to visual observation data, although the magnitude of the difference was smaller, and 95% confidence intervals overlapped for both species (Fig. 2).

## Discussion

The success of eDNA surveys depend on the development and validation of the assay, which involves collecting and filtering water samples, filter extraction, primer design, PCR optimization, and rigorous testing at different levels to avoid both false positive and false negative results^52^. eDNA assays that target mitochondrial DNA are generally preferred over those targeting nuclear DNA when available DNA is limited and/or degraded^53–57^. Primers amplifying short fragments are considered more suitable when the target DNA is degraded or in low concentrations, which is typical of DNA shed into the environment^58–61^. Multiple primers were designed, and selection was made based on primer specificity and sensitivity after conducting laboratory and field tests; we excluded any primers that did not amplify target DNA or if they amplified non-target DNA. Final primers successfully amplified a portion of the mitochondrial 16S rRNA gene for both *A. hazarensis* and *N. vicina* (50–150 bp). Our newly designed eDNA assay joins numerous others studies that have been successful in detecting a number of rare and cryptic species, including the Pool Frog (*Pelophylex lessonae*)^47^, Golden Tree Frog (*Phytotriades auratus*)^62^, Great Crested Newt (*Triturus cristatus*)^63^, and Idaho Giant Salamander (*Dicamptodon aterrimus*)^64^, and provides a framework for future eDNA sampling. Though eDNA assays can successfully detect a number of target species, other survey methods may still yield better detections. For example, Northern Leopard Frogs (*Rana pipiens*) are best detected using visual sightings in the summer and Boreal Chorus Frogs (*Pseudacris maculata*) by their breeding calls in the spring in southern Alberta, Canada^65^. Therefore, it remains important to compare eDNA assays to traditional methods to determine the efficiency of each method in detecting specific species.

It is unlikely that all individuals, populations, or species are ever detected**.** Detection of amphibians in streams is sometimes difficult when using visual encounter surveys due to complex topography, vegetation, water turbidity, flow rate, the cryptic nature of the species, and the low density of individuals^66–68^. High sensitivity is perceived to be a key advantage of species-specific eDNA methods^38,69–71^. Sensitivity in this context is defined as the probability of detecting target DNA at a site where that DNA is present. Our results showed that an individual’s ability to detect *A. hazarensis* through direct observation was low (~ 50%;  95% CI: 0.40–0.68), but it was high when using the eDNA assay (> 80%; 95% CI: 0.78–0.90), though the difference between the two methods was marginal for *N. vicina *(direct observation: > 65%; 95% CI: 0.56–0.78 vs. eDNA assay: > 75%; 95% CI: 0.73–0.82). The lower detection probabilities from visual observation data are likely due to the natural history of these species. Both *A. hazarensis* and *N. vicina* are nocturnal and inhabit montane freshwater streams, often seeking refuge under large rocks and boulders^72^. Even if individuals are not visually detected, they can still be detected through our eDNA assays. Additionally, detection of *N. vicina* was higher than *A. hazarensis* due to their large body size^72^ and the presence of *N. vicina* tadpoles throughout the year. In sum, our data show that eDNA assays outperform visual encounter surveys in detecting both *A. hazarensis* and *N. vicina*, which matches numerous other researchers who found that eDNA assays outperformed traditional methods of species detection^73–75^. These results help provide insight into monitoring endemic species, which are expected to occur in large numbers, yet may go undetected using traditional methods.

Our model identified the significance of temperature for the detection of *A. hazarensis* and *N. vicina* through visual observations, which matches other studies identifying temperature as a key covariate for the detection of amphibian species^36,49^. However, none of the studied parameters had any effect on the detection of *A. hazarensis* and *N. vicina* using eDNA. These results suggest that individuals may be more visible when temperatures are favorable, that they remain hidden under boulders when temperatures are not ideal, and that eDNA assays perform well in detecting these species regardless of temperature, which allows for a greater window in which eDNA sampling or monitoring could occur.

Using visual observation data, our occupancy model showed significant effects of elevation on *A. hazarensis* and *N. vicina*; with eDNA data, our occupancy model showed significant effects of both elevation and wetland type. Both species had a high relative occupancy at seasonal streams and springs compared to permanent streams based on eDNA data. Previously, elevation and wetland type were identified as strongly influencing the likelihood of occurrence of *A. hazarensis* and *N. vicina*, with both species showing a high likelihood of occurrence at sites with permanent water bodies^76^. We attribute this difference to the previous study only using visual observations, not eDNA detections. This difference between studies suggests that visual surveys may misinterpret species habitat use due to reduced detection abilities compared to eDNA surveys, particularly at seasonal stream and spring sites, and further highlights the utility of eDNA surveys for cryptic species.

We were also curious if the presence of one species influences the presence of another. To do so, we used a previously developed model which estimates the probability of occupancy for a subordinate species conditional upon the presence of a dominant species^77^. Like others who found no conditional or competitive interactions between sympatric species^77,78^, we also obtained similar results for our two frog species. The occurrence of *A. hazarensis* was not conditional upon *N. vicina* and vice versa. Since we assumed an asymmetric interaction between *A. hazarensis* and *N. vicina*, we suggest conducting multi-species (more than two interacting species) studies in future, as multi-species models have suggested that the probability of some pairs of species occupying the same site may vary along environmental gradients^79^.

## Conclusion

The development and implementation of this assay was successful at detecting *A. hazarensis* and *N. vicina* eDNA from field samples using primers targeting the mitochondrial 16S rRNA gene. We conducted 442 visual encounter and eDNA surveys across 34 sites for two years. During the visual encounter surveys, *A. hazarensis* was observed 44 times and *N. vicina* was observed 152 times. Using our newly designed eDNA assay, *A. hazarensis* was detected 196 times and *N. vicina* was detected 216 times. Estimates of *N. vicina* visual detection probability were greater than *A. hazarensis*, likely due to the species’ larger size and the presence of tadpoles year-round; however, detection probability from eDNA assays was high and similar for both species. Temperature was found to influence detection of *A. hazarensis* and *N. vicina* in visual encounter surveys, but not for eDNA surveys. Accurately locating populations is essential to understanding the current distribution of species and to guide management efforts. The results of this study show the efficacy of these eDNA assays in detecting both *A. hazarensis* and *N. vicina* independent of factors which affect species detection during visual encounter surveys. Both *A. hazarensis* and *N. vicina* occupy montane streams where individuals frequently seek refuge under large rocks. As a result, individuals are challenging to observe, resulting in false negatives during visual encounter surveys; however, our eDNA surveys continue to be able to successfully detect species in these habitats. Though they cannot fully eliminate the need for visual encounter surveys, eDNA surveys hold the potential to help fill knowledge gaps about the distribution of species, especially in habitats where detection of individuals is challenging.

## Materials and methods

### Species description

Asian spiny frogs (genera *Allopaa*, *Nanorana*, *Quasipaa,* and *Chaparana*) belong to the tribe Paini, family Dicroglossidae, and are endemic to south and southeast Asia. These anurans are found in montane, swift, boulder-strewn streams of southern China, Indochina, and the Himalayas^80,81^. The Hazara Torrent Frog (*Allopaa hazarensis*; Dubois and Khan, 1979) is endemic to the springs and streams of northern Pakistan. The frog has a head longer than it is wide, a naris above the canthus, and a clear gray transverse band on the back of the head joining the posterior borders of the eyelids^72^. The Murree Hills Frog (*Nanorana vicina*; Stoliczka, 1872) is endemic to southern Asia (Pakistan, India). The tympanum is indistinct; the head is wider than it is long; and the canthus is round. The frog has its naris under the canthus, closer to eye than snout tip, and a thick fold of skin from back of upper eyelid to the angle of jaws^82^.

### Study area

**Figure 3:**
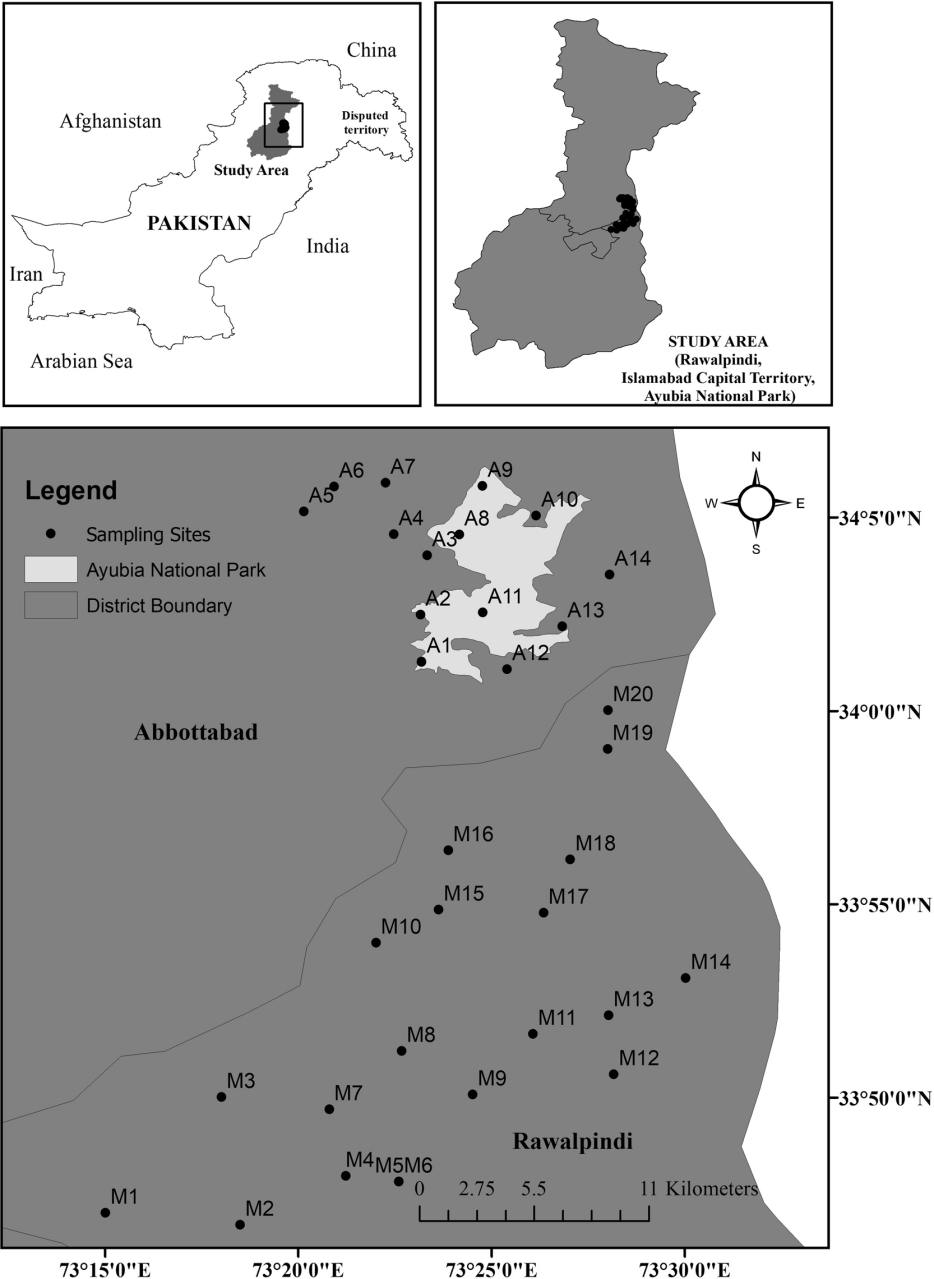
Map of Pakistan indicating study sites of Tehsil Murree (M1–M20), located in the Rawalpindi District, Punjab Province, and those of Ayubia National Park (A1–A14), located in Abottabad Distrct, Khyber Pakhtunkhwa Province. Map generated using ArcMap 10.4.1 (/www.esri.com/en-us/arcgis/products/arcgis-desktop/resources).

We conducted the present study in two regions: (1) Tehsil Murree (33.90°N, 73.39°E), located in Rawalpindi District, Punjab Province (n = 20 sites; M1–M20; Fig. 3) and (2) Ayubia National Park (34.07°N, 73.38°E), located in Abottabad District, Khyber Pakhtunkhwa Province (n = 14 sites; A1–A14; Fig. 3). Tehsil Murree is located in the foothills of the Himalayas, comprising an area of 69,750 ha at 804–2,291 m elevation^83^. Climate of the area is subtropical highland^84^ with mean maximum temperature of 25 °C and annual precipitation of 1789 mm^83^. The habitat types of the area are Sub-tropical Chir Pine Forest and Himalayan Moist Temperate Forest^85^. Ayubia National Park comprises an area of 3312 ha, situated at an elevation of 2300–3000 m. Climate of the area is temperate^84^, with a mean maximum temperature of 19.2 °C and annual precipitation of 1412 mm. The park has three types of forest: Sub-alpine Meadows, Sub-tropical Chir Pine Forest, and Himalayan Moist Temperature Forest^86^.

### Sequencing of the 16S rRNA mitochondrial gene

We amplified the mitochondrial 16S rRNA gene by using previously reported universal primers 16Sar (5'-CGCCTGTTTAYCAAAAACAT-3') and 6Sbr (5'-CCGGTYTGAACTCAGATCAYGT-3')^87^, which have been suggested as universal markers to barcode amphibians for species determination^88^. DNA was extracted using QIAGEN DNeasy Blood and Tissue Kits (QIAGEN, Germany, Cat #69504) following the manufacturer’s protocol. Genomic DNA concentrations were determined for all extracts using a Qubit Fluorometer (Life Technologies, OR, USA). Real-time polymerase chain reaction (qPCR) was used to amplify a fragment of the mitochondrial 16S rRNA gene with the following thermal cycling profile: 10 min at 95 °C (initial denaturing) followed by 40 cycles at 95 °C for 30 s (denaturing), 60 °C for 60 s (annealing), and 72 °C for 60 s (extension). The amplicon size of amplified DNA was assessed by gel electrophoresis using a 2% molecular grade agarose gel with a run time of 50 min at 90 V. PCR products were purified using ExoSAP-IT PCR Product Cleanup Reagent (Thermo Fisher Scientific, Lithuania, Cat #78200) and Sanger sequenced at the Research Technology Service Facility of Michigan State University.

### Design and validation of species-specific primers

A set of species-specific primer sets (n = 5 for *A. hazarensis*; n = 14 for *N. vicina*) were designed to target a small region of the mitochondrial 16S rRNA gene. The sequence information for these genes was obtained for *A. hazarensis* and *N. vicina* by Sanger sequencing. Primers were designed using Primer Blast with default settings, except the PCR product size, which was maintained between 50 and 150 bp. All primer sets were evaluated for their amplification efficiency and performance using tissue-derived DNA from *A. hazarensis* and *N. vicina*. The specificity and sensitivity of these primer sets was checked at three stages: in silico, in vitro, and in situ. For the in silico check of the designed primers for non-specific targets, all sequences were evaluated using the Basic Local Alignment Search Tool (BLAST) to confirm their percent sequence similarity with other species (http://blast.ncbi.nlm.nih.gov/). To our knowledge, the mitochondrial 16S RNA gene sequence of *A. hazarensis* and *N*. *vicina* themselves are novel and have not been previously reported. The sequences were aligned by using software MEGA X (Molecular Evolutionary Genetics Analysis). For in vitro testing, DNA extracted from tissues of target and non-target species were checked for primer specificity. For in situ analysis, assays were applied to eDNA samples from habitats where *A. hazarensis* and *N*. *vicina* were known to be present and where the species were known to be absent to check specificity and sensitivity of the eDNA assay in natural conditions. A serial dilution was made (10 to 0.001) to assess the analytical sensitivity of primer sets and the threshold cycle time (C_t_) was obtained for the series.

### Sampling sites

The study area was stratified based on elevation (700–3000 m), forest types (Sub-tropical Broad-leaved Evergreen Forest, Sub-tropical Chir Pine Forest, and Himalayan Moist Temperature Forest) and wetland types (permanent streams [n = 24], seasonal streams [n = 4], springs [n = 5]). For an individual stream site, 7 pools (situated a minimum distance of 250 m from each other) were surveyed. For occupancy modeling, we used site covariates such as wetland type, elevation (m), species (*A. hazarensis* and *N. vicina*), and year of survey (February–June 2017 = year 1; February–June 2018 = year 2), and we used air temperature (°C) as a visit covariate (Supplementary material 2 & 3).

### eDNA sample collection and analysis

At each site, seven 1-L water samples were collected in polypropylene bottles. During transport the samples were stored in a cooler with ice packs. Filtration of all water samples was carried out within 24 h of collection using 0.45-μm pore size cellulose nitrate filter (47-mm diameter, Sartorius Stedim Biotech AGGöttingen, Germany), sterile preloaded disposable filter cup and a hand-held vacuum pump. The filters were stored in 95% ethanol in a 2-ml vial at room temperature until DNA was extracted using a previously described protocol^40^.

To avoid contamination at any stage of the procedure (from sample collection to amplification of DNA), our tools were sterilized (autoclaved) and benchtops were rinsed with alcohol. Filter papers were removed from ethanol after transfer to the lab, air-dried using forceps, and halved. One-half of the filter was used for DNA extraction while second part was stored in 95% ethanol. Total DNA was recovered from each filtered sample using the QIAGEN DNeasy Power Water kit (QIAGEN, Germany, Cat #14900-50) according to the manufacturer’s protocol. Extracted DNA was stored at − 20 °C until analyzed further by real-time quantitative PCR (qPCR). For *A. hazarensis*, primers targeting mitochondrial 16S rRNA gene (Allopaa4-F [TAG CAC GAA AAT TCT GCC TGT], Allopaa4-R [TCC GTT CAT CGT GGA GGT TTA]; targeting ~ 50 bp) and for *N. vicina* (Nanorana8-F [ATT CTT AGG GTG GTG GCC CT, Nanorana8-R [ATT CTG CAT AGC CTG GGG TC]; targeting ~ 90 bp) were used. Both primer sets were developed and validated as part of this study.

After initial optimization, all qPCR analyses were carried out in a final volume of 25 µl, consisting of 1 µl of DNA extract as template, 12.5 µL of Power SYBR Green (Applied Biosystems, UK), 1.25 µl of primer mix (10 µM), and 10.25 µl of nuclease-free water and a Mastercycler ep realplex2 (Eppendorf, Hamburg, Germany). Each DNA sample was analyzed in triplicate. A negative control (nuclease-free water) as well as positive control (tissue DNA from *A. hazarensis* and *N. vicina*) were included during qPCR. A sample was considered eDNA-positive if at least two of the three technical replicates amplified, the positive control amplified (indicating the qPCR reactions successfully amplified), and the negative control had no amplification (indicating no laboratory contamination).

### Comparing visual observation data and eDNA data

We used visual encounter surveys to document the presence of *A. hazarensis* and *N. vicina* at each sampling site. The observers actively and thoroughly searched the sampling sites for a pre-defined time (30 min) during evening and at night to record species, sex, and life stage (tadpole, sub-adult, adult)^89^. We then compared our data on detection through eDNA and direct sighting by site occupancy and detection probabilities of the two species.

### Occupancy modeling

We were interested in the occupancy of each species and also the possibility of interactions between the two species. Thus, we fit separate multispecies occupancy models^79^ to the visual observation data and the eDNA data to account for imperfect detection. Multispecies occupancy models allow for simultaneous modeling of covariates impacting occupancy and detection probability of individual species as well as covariates that influence the interaction of the species^79^. The multispecies model allowed us to assess if the presence of one species affected the occupancy of the other and vice-versa. For a two-species model, three occupancy-related natural parameters are estimated: two parameters associated with occupancy for each individual species, and a parameter associated with their interaction^79^. We applied the same two-stage modeling process to each data type. In the first stage, for the visual observation data, we identified four candidate models for the detection probability component of the model: (1) a null model, (2) year, (3) temperature during sampling, and (4) year and temperature. We fit the four models, maintaining a constant null model (without a two-species interaction) for occupancy, and selected the top-ranked detection model based on Akaike Information Criterion (AIC) to use in the second modeling stage. We used the same approach for the eDNA data except we did not include temperature as a covariate, resulting in a set of two candidate models.

For the second stage of modeling, we compared ten candidate models for the occupancy process. These models were the same for both data types. The first five candidate models included: (1) a null model, (2) year, (3) elevation and year, (4) wetland type and year, and (5) elevation, wetland type, and year. These covariates were applied to the single-species occupancy natural parameters associated with the two species. The interaction term for these five models was fixed at 0, implying no interaction between the two species. The second five candidate models included identical combinations of covariates, but for these models the covariates were also applied to the two-species interaction term. We ranked the ten candidate models using AIC and selected the top model for further inference. With this approach, we were able to identify which covariates affect species occupancy and if there was support for an interaction between the two species (Supplementary material 1).

We fit and ranked all candidate models in R^90^ using the unmarked package^51^. For both data types, initial modeling revealed problems with boundary estimates^91^. To address this, we fit all models using a penalized likelihood approach^91,92^.

## Supplementary Information

Supplementary Material 1.

Supplementary Material 2.

Supplementary Material 3.

## Data Availability

The authors have archived the data with Dryad (10.5061/dryad.8sf7m0cqw).

## Code availability

The R code are available at Dryad (10.5061/dryad.8sf7m0cqw).
