# Supplementary material for: Development and validation of an eDNA protocol for monitoring endemic Asian spiny frogs in the Himalayan region of Pakistan
Source: Sci Rep. 2022 Apr 4;12:5624. doi: 10.1038/s41598-022-09084-1 (PMC8979970; doi:10.1038/s41598-022-09084-1)
Supplement: Supplementary file 1 — Supplementary Material 1. [file 41598_2022_9084_MOESM1_ESM.pdf]

Code for:

Development and validation of an eDNA protocol for monitoring endemic Asian spiny frogs in the Himalayan region of Pakistan

Saeed et al. (2022) Scientific Reports

Written by Ken Kellner

# Load required libraries

```
library(tidyverse)

library(readxl)

library(unmarked)
```

# Function to process dates in raw data

```
convert_dates <- function(date_raw){

  date_raw <- gsub("/","-",date_raw)

  out <- rep(NA, length(date_raw))

  out <- as.Date(out)

  good_date <- grepl("2017|2018", date_raw)

  out[good_date] <- as.Date(date_raw[good_date], "%d-%m-%Y")

  temp <- as.character(as.Date(as.integer(date_raw[!good_date]), origin = "1899-12-30"))

  temp <- as.Date(temp, format="%Y-%d-%m")

  out[!good_date] <- temp

  out

}
```

# ggplot theme code

```
mytheme <- theme_bw(base_size=14) +

  theme(panel.grid=element_blank())
```

# Visual detection data--

### Data setup

#Read in the data from visual detection

```
alha_ves <- read_excel('Allopaa hazarensis.xlsx', sheet='1-AH_VES_Detection',

                    skip=1) %>%
```

```
  select(-P.Stream, -Spring, -S.Stream, -EBLF, -STPF, -HMTF, -VES, -eDNA) %>%
  rename(Elevation=`Elevation(m)`) %>%
  select(-...38, -...39, -...40, -...41)
nana_ves <- read_excel('Nanorana vicina.xlsx', sheet='1-NV_VES_Detection',
                   skip=1) %>%
  select(-P.Stream, -Spring, -S.Stream, -EBLF, -STPF, -HMTF, -VES, -eDNA) %>%
  rename(Elevation=`Elevation(m)`) %>%
  select(-...38, -...39, -...40)
date_raw <- names(read_excel('Allopaa hazarensis.xlsx',
                             sheet='1-AH_VES_Detection'))[17:37]
alha_ves_date <- convert_dates(date_raw)
```

# Temperature

```
alha_temp <- read_excel('Allopaa hazarensis.xlsx', sheet='5-Visit_ovariate',
                   skip=1) %>%
  select(-Tehsil,-Location) %>%
  select(-...25, -...26, -...27)
temp_dates <- names(read_excel('Allopaa hazarensis.xlsx', sheet='5-Visit_ovariate'))[4:24]
temp_dates <- convert_dates(temp_dates)
```

# Site covariates

```
site_covs <- alha_ves %>%
  dplyr::select(ID:Forest) %>%
  mutate(Wetlands=factor(Wetlands), Forest=factor(Forest)) %>%
  as.data.frame()
```

# obs covariates

```
obs_covs <- list(temp=alha_temp %>% select(-ID))
obs_covs$temp <- cbind(obs_covs$temp, matrix(NA, nrow(site_covs), 5))
```

# presence/absence matrices

```
y <- as.matrix(alha_ves %>% select(VES1:VES21))
```

```
y1 <- y[,1:7]

y2 <- cbind(y[,14:19],NA)

y <- rbind(y1,y2)

y_nana <- as.matrix(nana_ves %>% select(VES1:VES21))

y1 <- y_nana[,1:7]

y2 <- cbind(y_nana[,14:19],NA)

y_nana <- rbind(y1,y2)


oc1 <- obs_covs$temp[,1:7] # through june

oc2 <- cbind(obs_covs$temp[,14:19],NA) #through june

names(oc2) <- names(oc1)

#Construct unmarkedFrame

umf <- unmarkedFrameOccuMulti(y=list(Alha=y, Nana=y_nana),

                    siteCovs=rbind(site_covs, site_covs),

                    obsCovs=list(temp=as.matrix(rbind(oc1,oc2))))

umf@siteCovs$year <- factor(c(rep("1", nrow(site_covs)), rep("2", nrow(site_covs))))


### Fit models

# Do this in two stages, starting with detection.

sf <- c("~1","~1","0")

df <- rep("~1", 2)

mod_null <- occuMulti(df, sf, umf)

mod_temp <- occuMulti(rep("~scale(temp)",2), sf, umf)

mod_year <- occuMulti(rep("~year",2), sf, umf)

mod_temp_year <- occuMulti(rep("~scale(temp) + year",2), sf, umf)

# Table 2 part 1

modSel(fitList(mod_null,mod_temp, mod_year, mod_temp_year))


#Best detection model included temperature and year for both species.
```

```
df <- rep("~scale(temp)+year", 2)
```

# Fit various occupancy models with penalty term

# to handle boundary estimates

```
forms <- list(null_noint=c("~1","~1","0"), null_int=rep("~1",3),
        year_noint=c("~year","~year","0"), year_int=rep("~year",3),
        el_yr_noint=c(rep("~scale(Elevation)+year",2),"0"), el_yr_int=c(rep("~scale(Elevation)+year",3)),
        wet_yr_noint=c(rep("~Wetlands+year",2),"0"), wet_yr_int=rep("~Wetlands+year",3),
        el_wet_yr_noint=c(rep("~scale(Elevation)+Wetlands+year",2),"0"),
        el_wet_yr_int=c(rep("~scale(Elevation)+Wetlands+year",3)))


umf_sub <- umf[which(umf@siteCovs$Wetland!="Seasonal stream"),]

umf_sub@siteCovs$Wetlands=droplevels(umf_sub@siteCovs$Wetland)
```

#set.seed(123)

#pmod <- occuMulti(df, forms[[length(forms)]], umf)

#pmod <- optimizePenalty(pmod)

```
mods <- lapply(forms, function(x) occuMulti(df, x, umf, penalty=2))
```

# Table 3 part 1

```
modSel(fitList(fits=mods))
```

#Best occupancy model included elevation and year.

# Table 4

```
top <- mods$el_yr_noint

summary(mods$el_yr_noint)
```

# mean detection by species and year

```
nd_det <- data.frame(temp=mean(top@data@obsCovs$temp, na.rm=T), year=factor(c("1","2"),
levels=c("1","2")))

predict(top, 'det', newdata=nd_det)
```

# Calculate occupancy each species and year using empirical Bayes methods

# using top model

```
nsims <- 100
mu <- coef(top)
Sigma <- vcov(top)

set.seed(123)
bs <- MASS::mvrnorm(nsims, mu, Sigma)

M <- numSites(top@data)
zpost <- matrix(NA, nrow=M, ncol=nsims)
mod_star <- top
```

# A. hazarensis

```
no_detect_alha <- apply(top@data@ylist$Alha, 1, function(x) 1-max(x, na.rm=T))

pb = txtProgressBar(min = 0, max = nsims, initial = 0)
for (i in 1:nsims){

  mod_star@estimates@estimates$state@estimates <- bs[i, 1:6]
  mod_star@estimates@estimates$det@estimates <- bs[i, 7:12]

  nul <- capture.output(psi <- predict(mod_star, 'state', species='Alha', se=FALSE)$Predicted)

  p <- getP(mod_star)$Alha
  qT <- apply(p, 1, function(x) prod(1-x, na.rm=T))

  for (j in 1:M){
    if(no_detect_alha[j] == 0){ #If detected at least once at site j
```

```
    zpost[j,i] <- 1
   } else{ #If never detected at site j
    psi_con <- psi[j] * qT[j] / (psi[j] * qT[j] + (1-psi[j]))
    zpost[j,i] <- rbinom(1, 1, psi_con)
   }
  }
  setTxtProgressBar(pb,i)
}
close(pb)

hist(apply(zpost, 2, mean), xlim=c(0,0.6))
abline(v=mean(1-no_detect_alha), col='red')

yr1 <- top@data@siteCovs$year=="1"
post_yr1 <- apply(zpost[yr1,], 2, mean)
naive_yr1 <- mean(1-no_detect_alha[yr1])

mean(post_yr1)
sd(post_yr1)
quantile(post_yr1, c(0.025, 0.975))

hist(post_yr1, xlim=c(0,0.6))
abline(v=naive_yr1, col='red')

yr2 <- top@data@siteCovs$year=="2"
post_yr2 <- apply(zpost[yr2,], 2, mean)
naive_yr2 <- mean(1-no_detect_alha[yr2])

mean(post_yr2)
```

```
sd(post_yr2)
quantile(post_yr2, c(0.025, 0.975))

hist(post_yr2, xlim=c(0,0.6))
abline(v=naive_yr2, col='red')
```

# N. vicina

```
set.seed(123)
no_detect_nana <- apply(top@data@ylist$Nana, 1, function(x) 1-max(x, na.rm=T))

pb = txtProgressBar(min = 0, max = nsims, initial = 0)
for (i in 1:nsims){

  mod_star@estimates@estimates$state@estimates <- bs[i, 1:6]
  mod_star@estimates@estimates$det@estimates <- bs[i, 7:12]

  nul <- capture.output(psi <- predict(mod_star, 'state', species='Nana', se=FALSE)$Predicted)

  p <- getP(mod_star)$Nana
  qT <- apply(p, 1, function(x) prod(1-x, na.rm=T))

  for (j in 1:M){
    if(no_detect_nana[j] == 0){ #If detected at least once at site j
      zpost[j,i] <- 1
    } else{ #If never detected at site j
      psi_con <- psi[j] * qT[j] / (psi[j] * qT[j] + (1-psi[j]))
      zpost[j,i] <- rbinom(1, 1, psi_con)
    }
  }
```

```
  setTxtProgressBar(pb,i)
}
close(pb)

hist(apply(zpost, 2, mean), xlim=c(0,0.6))
abline(v=mean(1-no_detect_nana), col='red')

yr1 <- top@data@siteCovs$year=="1"
post_yr1 <- apply(zpost[yr1,], 2, mean)
naive_yr1 <- mean(1-no_detect_nana[yr1])

mean(post_yr1)
sd(post_yr1)

hist(post_yr1, xlim=c(0,0.6))
abline(v=naive_yr1, col='red')

yr2 <- top@data@siteCovs$year=="2"
post_yr2 <- apply(zpost[yr2,], 2, mean)
naive_yr2 <- mean(1-no_detect_nana[yr2])

mean(post_yr2)
sd(post_yr2)

hist(post_yr2, xlim=c(0,0.6))
abline(v=naive_yr2, col='red')
```

# eDNA Detections--

### Data Setup

```
alha_dna <- read_excel('Allopaa hazarensis.xlsx', sheet='2-AH_eDNA-Detection',
                   skip=1) %>%
  select(-VES, -eDNA) %>%
  rename(Elevation=`Elevation(m)`) %>%
  select(-...30, -...31, -...32)
```

# y matrix

```
y_dna <- as.matrix(alha_dna %>% select(eDNA1:eDNA21))
y1 <- y_dna[,c(1:7)]
y2 <- cbind(y_dna[,14:19], NA)
y_alha <- rbind(y1,y2)
nana_dna <- read_excel('Nanorana vicina.xlsx', sheet='2-NV_eDNA detection',
                   skip=1) %>%
  select(-VES, -eDNA) %>%
  rename(Elevation=`Elevation(m)`) %>%
  select(-...30, -...31, -...32)
```

# y matrix

```
y_dna <- as.matrix(nana_dna %>% select(eDNA1:eDNA21))
y1 <- y_dna[,c(1:7)]
y2 <- cbind(y_dna[,14:19], NA)
y_nana <- rbind(y1,y2)
```

# make unmarkedFrame

```
umf_dna <- unmarkedFrameOccuMulti(y=list(Alha=y_alha, Nana=y_nana),
                  siteCovs=rbind(site_covs, site_covs),
                  obsCovs=NULL)
umf_dna@siteCovs$year <- factor(c(rep("1", nrow(site_covs)), rep("2", nrow(site_covs))))
```

### Fit Models

# Pick detection model

```
sf <- c("~1","~1","0")

df <- rep("~1", 2)


mod_null <- occuMulti(df, sf, umf_dna)

mod_year <- occuMulti(rep("~year",2), sf, umf_dna)
```

# Table 2 part 2

```
modSel(fitList(mod_null, mod_year))
```

# Best detection model had no covariates.

# Pick occupancy model

```
df <- rep("~1", 2)


forms <- list(null_noint=c("~1","~1","0"), null_int=rep("~1",3),
        year_noint=c("~year","~year","0"), year_int=rep("~year",3),
        el_yr_noint=c(rep("~scale(Elevation)+year",2),"0"), el_yr_int=c(rep("~scale(Elevation)+year",3)),
        wet_yr_noint=c(rep("~Wetlands+year",2),"0"), wet_yr_int=rep("~Wetlands+year",3),
        el_wet_yr_noint=c(rep("~scale(Elevation)+Wetlands+year",2),"0"),
        el_wet_yr_int=c(rep("~scale(Elevation)+Wetlands+year",3)))
```

#set.seed(123)

#pmod <- occuMulti(df, forms[[length(forms)]], umf_dna)

#pmod <- optimizePenalty(pmod)

```
mods_dna <- lapply(forms, function(x) occuMulti(df, x, umf_dna, penalty=0.5))
```

# Table 3 part 2

```
modSel(fitList(fits=mods_dna))
```

#Top model included elevation, wetland type and year.

# Again, no interaction term in the best model.

# Table 5

```
top_dna <- mods_dna$el_wet_yr_noint

summary(mods_dna$el_wet_yr_noint)
```

# Average detection by species

```
nd_det_dna <- data.frame(year=factor(c("1","2"), levels=c("1","2")))

predict(top_dna, 'det', newdata=nd_det_dna)
```

# Occupancy estimates for each species and year using empirical Bayes methods

```
nsims <- 100

mu <- coef(top_dna)

Sigma <- vcov(top_dna)


set.seed(123)

bs <- MASS::mvrnorm(nsims, mu, Sigma)


M <- numSites(top@data)

zpost <- matrix(NA, nrow=M, ncol=nsims)

mod_star <- top_dna
```

# A. hazarensis

```
no_detect_alha <- apply(top_dna@data@ylist$Alha, 1, function(x) 1-max(x, na.rm=T))


pb = txtProgressBar(min = 0, max = nsims, initial = 0)

for (i in 1:nsims){


  mod_star@estimates@estimates$state@estimates <- bs[i, 1:10]
```

```
  mod_star@estimates@estimates$det@estimates <- bs[i, 11:12]

  nul <- capture.output(psi <- predict(mod_star, 'state', species='Alha', se=FALSE)$Predicted)

  p <- getP(mod_star)$Alha
  qT <- apply(p, 1, function(x) prod(1-x, na.rm=T))

  for (j in 1:M){
    if(no_detect_alha[j] == 0){ #If detected at least once at site j
      zpost[j,i] <- 1
    } else{ #If never detected at site j
      psi_con <- psi[j] * qT[j] / (psi[j] * qT[j] + (1-psi[j]))
      zpost[j,i] <- rbinom(1, 1, psi_con)
    }
  }
  setTxtProgressBar(pb,i)
}
close(pb)

hist(apply(zpost, 2, mean), xlim=c(0,1))
abline(v=mean(1-no_detect_alha), col='red')

yr1 <- top_dna@data@siteCovs$year=="1"
post_yr1 <- apply(zpost[yr1,], 2, mean)
naive_yr1 <- mean(1-no_detect_alha[yr1])

mean(post_yr1)
sd(post_yr1)
quantile(post_yr1, c(0.025, 0.975))
```

```
hist(post_yr1, xlim=c(0,0.6))
abline(v=naive_yr1, col='red')

yr2 <- top_dna@data@siteCovs$year=="2"
post_yr2 <- apply(zpost[yr2,], 2, mean)
naive_yr2 <- mean(1-no_detect_alha[yr2])

mean(post_yr2)
sd(post_yr2)
quantile(post_yr2, c(0.025, 0.975))

hist(post_yr2, xlim=c(0,0.6))
abline(v=naive_yr2, col='red')
```

# N. vicina

```
set.seed(123)
no_detect_nana <- apply(top_dna@data@ylist$Nana, 1, function(x) 1-max(x, na.rm=T))

pb = txtProgressBar(min = 0, max = nsims, initial = 0)
for (i in 1:nsims){

  mod_star@estimates@estimates$state@estimates <- bs[i, 1:10]
  mod_star@estimates@estimates$det@estimates <- bs[i, 11:12]

  nul <- capture.output(psi <- predict(mod_star, 'state', species='Nana', se=FALSE)$Predicted)

  p <- getP(mod_star)$Nana
  qT <- apply(p, 1, function(x) prod(1-x, na.rm=T))
```

```
  for (j in 1:M){
    if(no_detect_nana[j] == 0){ #If detected at least once at site j
      zpost[j,i] <- 1
    } else{ #If never detected at site j
      psi_con <- psi[j] * qT[j] / (psi[j] * qT[j] + (1-psi[j]))
      zpost[j,i] <- rbinom(1, 1, psi_con)
    }
  }
  setTxtProgressBar(pb,i)
}
close(pb)

hist(apply(zpost, 2, mean), xlim=c(0,0.6))
abline(v=mean(1-no_detect_nana), col='red')

yr1 <- top_dna@data@siteCovs$year=="1"
post_yr1 <- apply(zpost[yr1,], 2, mean)
naive_yr1 <- mean(1-no_detect_nana[yr1])

mean(post_yr1)
sd(post_yr1)

hist(post_yr1, xlim=c(0,0.6))
abline(v=naive_yr1, col='red')

yr2 <- top_dna@data@siteCovs$year=="2"
post_yr2 <- apply(zpost[yr2,], 2, mean)
naive_yr2 <- mean(1-no_detect_nana[yr2])
```

```
mean(post_yr2)
sd(post_yr2)

hist(post_yr2, xlim=c(0,0.6))
abline(v=naive_yr2, col='red')
```

# Figures---

```
nd_base <- data.frame(Elevation=mean(siteCovs(umf)$Elevation), Wetlands="Permanent stream",
        year="1", temp=mean(obsCovs(umf)$temp, na.rm=T))
```

# Figure 1

```
elev_seq <- seq(min(siteCovs(umf)$Elevation), max(siteCovs(umf)$Elevation), length.out=100)
nd <- nd_base[rep(1,100),]
nd$Elevation <- elev_seq

pr_ves_alha <- predict(top, type='state', newdata=nd, species='Alha', nsim=5000)
pr_ves_alha$method <- 'Visual'
pr_ves_alha$species <- 'Allopaa hazarensis'

pr_dna_alha <- predict(top_dna, type='state', newdata=nd, species='Alha',nsim=5000)
pr_dna_alha$method <- 'eDNA'
pr_dna_alha$species <- 'Allopaa hazarensis'

pr_ves_nana <- predict(top, type='state', newdata=nd, species='Nana',nsim=5000)
pr_ves_nana$method <- 'Visual'
pr_ves_nana$species <- 'Nanorana vicina'

pr_dna_nana <- predict(top_dna, type='state', newdata=nd, species='Nana',nsim=5000)
```

```
pr_dna_nana$method <- 'eDNA'
pr_dna_nana$species <- 'Nanorana vicina'

pldat <- rbind(pr_ves_alha, pr_dna_alha, pr_ves_nana, pr_dna_nana)
pldat$elev <- rep(elev_seq,4)

elev_plot <- pldat %>%
  mutate(method=factor(method, levels=c("Visual","eDNA"))) %>%
  ggplot(aes(x=elev, y=Predicted)) +
  geom_ribbon(aes(ymin=lower,ymax=upper, fill=method), alpha=0.3) +
  geom_line(aes(col=method)) +
  facet_wrap("species") +
  mytheme +
  labs(x="Elevation (m)", y="", fill="Method",
       col="Method") +
  theme(legend.position=c(0.4,0.82),
        legend.title=element_text(size=12),
        legend.background=element_blank(),
        axis.title.y=element_blank(),
        strip.background=element_rect("white"), strip.text=element_text(face="italic")) +
  ylim(0,1)

nd <- nd_base[rep(1,3),]
nd$Wetlands <- factor(levels(siteCovs(umf)$Wetland),levels=levels(siteCovs(umf)$Wetland))

pr_dna_alha <- predict(top_dna, type='state', newdata=nd, species='Alha',nsim=1000)
pr_dna_alha$method <- 'eDNA'
pr_dna_alha$species <- 'Allopaa hazarensis'
```

```
pr_dna_nana <- predict(top_dna, type='state', newdata=nd, species='Nana',nsim=1000)
pr_dna_nana$method <- 'eDNA'
pr_dna_nana$species <- 'Nanorana vicina'

pldat <- rbind(pr_dna_alha, pr_dna_nana)
pldat$Wetlands <- rep(nd$Wetlands,2)

wet_plot <- pldat %>%
  mutate(Wetlands=fct_recode(Wetlands, `Permanent\nstream`="Permanent stream",
        `Seasonal\nstream`="Seasonal stream")) %>%
  ggplot(aes(x=Wetlands, y=Predicted)) +
  geom_errorbar(aes(ymin=lower,ymax=upper), width=0.2) +
  geom_point(size=3) +
  facet_wrap("species") +
  mytheme +
  labs(x="Wetland type", y="") +
  theme(legend.position=c(0.4,0.7), legend.title=element_blank(),
        legend.background=element_blank(),
        axis.title.y=element_blank(),
        strip.background=element_rect("white"), strip.text=element_blank()) +
  ylim(0,1)

pl <- cowplot::plot_grid(elev_plot, wet_plot, ncol=1, rel_heights=c(1.05,1))

y.grob <- grid::textGrob("Occupancy and 95% CI",
                gp=grid::gpar(fontsize=14), rot=90)

tiff("Figure1.tiff", compression='lzw', height=7, width=7, res=300, units='in')
gridExtra::grid.arrange(gridExtra::arrangeGrob(pl, left = y.grob))
```

```
dev.off()

# Figure 2
prdat <- rbind(
  data.frame(Predicted=mean(apply(umf@ylist$Alha, 1, function(x) sum(x,na.rm=T)>0)),
                 SE=NA,lower=NA,upper=NA),
  data.frame(Predicted=mean(apply(umf_dna@ylist$Alha, 1, function(x) sum(x,na.rm=T)>0)),
                 SE=NA,lower=NA,upper=NA),
  predict(top, type='state', newdata=nd_base, species='Alha'),
  predict(top_dna, type='state', newdata=nd_base, species='Alha')
)
prdat$method <- c("Visual", "eDNA", "Visual", "eDNA")
prdat$type <- rep(c("Naive", "Model"),each=2)
prdat$species <- "Allopaa hazarensis"

prdat_nana <- rbind(
  data.frame(Predicted=mean(apply(umf@ylist$Nana, 1, function(x) sum(x,na.rm=T)>0)),
                 SE=NA,lower=NA,upper=NA),
  data.frame(Predicted=mean(apply(umf_dna@ylist$Nana, 1, function(x) sum(x,na.rm=T)>0)),
                 SE=NA,lower=NA,upper=NA),
  predict(top, type='state', newdata=nd_base, species='Nana'),
  predict(top_dna, type='state', newdata=nd_base, species='Nana')
)
prdat_nana$method <- c("Visual", "eDNA", "Visual", "eDNA")
prdat_nana$type <- rep(c("Naive", "Model"),each=2)
prdat_nana$species <- "Nanorana vicina"

pos=position_dodge(width=0.4)
```

```
occ_plot <- rbind(prdat, prdat_nana) %>%
  filter(type != "Naive") %>%
  mutate(method=factor(method, levels=c("Visual","eDNA"))) %>%
  ggplot(aes(x=method, y=Predicted)) +
  geom_errorbar(aes(ymin=lower,ymax=upper), width=0.2) +
  geom_point(size=3) +
  facet_wrap("species") +
  mytheme +
  labs(x="Data collection method", y="Occupancy and 95% CI") +
  theme(legend.position=c(0.1,0.9), legend.title=element_blank(),
        strip.background=element_rect("white"),
        legend.background=element_blank(),
        strip.text=element_blank())

nd_det <- data.frame(temp=mean(umf@obsCovs$temp,na.rm=T), year=factor(1,levels=c(1,2)))

prdet <- rbind(predict(top, type="det", newdata=nd_det, species="Alha"),
               predict(top_dna, type="det", newdata=nd_det, species="Alha"),
               predict(top, type="det", newdata=nd_det, species="Nana"),
               predict(top_dna, type="det", newdata=nd_det, species="Nana"))
prdet$method <- factor(rep(c("Visual", "eDNA"), 2),levels=c("Visual", "eDNA"))
prdet$species <- rep(c("Allopaa hazarensis","Nanorana vicina"),each=2)

det_plot <- prdet %>%
  ggplot(aes(x=method, y=Predicted)) +
  geom_errorbar(aes(ymin=lower,ymax=upper), width=0.2) +
  geom_point(size=3) +
  facet_wrap("species") +
  mytheme +
```

```
 labs(x="Data collection method", y="Detection and 95% CI") +
 theme(legend.position=c(0.1,0.9), legend.title=element_blank(),
       strip.background=element_rect("white"),
       legend.background=element_blank(),
       axis.title.x=element_blank(),
       axis.ticks.x=element_blank(),
       axis.text.x=element_blank(),
       strip.text=element_text(face="italic"))


cowplot::plot_grid(det_plot, occ_plot, ncol=1, rel_heights=c(1,1.1))

ggsave("Figure2.tiff", compression='lzw')
```
